# Typhus Fever, Treated by Brandy, &c.

**Published:** 1854-03

**Authors:** 


### *Typhus Fever, treated by free exhibition of Brandy, etc.*

BY DR. TODD.

We have recently watched, with great interest, a series of severe cases of typhus fever, under the care of Dr. Todd, in this hospital, in which an almost uniform plan of treatment, by means of the very free exhibition of stimulants, more especially brandy, has been resorted to with great succes. Reflecting instructively, as these cases do, on one of the most important questions in the whole range of practical medicine, we hasten to bring their chief features before the attention of our readers. The series consists of eighteen cases; and as we cannot, of course, find space for the details of the whole, we shall content ourselves by giving a brief synopsis of them. The whole having occurred within the last few months, and several of them within a few weeks, they present, we believe, fair specimens of the form of fever lately and still prevalent in the Metropolis. They do not, however, comprise all which have been under Dr. Todd's care during the time referred to, but only those of well-marked typhus type, and which agreed in presenting the following symptoms previous to the commencement of the treatment: A copious eruption of scattered measle-like spots (mulberry, or typhus rash;) bowels either confined or but slightly relaxed; great prostration of strength; delirium (in six cases coma was present;) a small and very rapid pulse. It may be well to premise, that they were treated as is done in almost all general hospitals in the open wards, their beds being purposely arranged so as to occur at some distance from each other, in order to prevent the accumulation of contagious emanations. The treatment pursued consisted in administering, either every hour or every half-hour, day and night, from half an ounce to an ounce of brandy, with a draught every second hour, containing sp. æth. chloric *m*x. ammonia carbonatis gr. v., aq. pur. ℥j. The patients were induced to drink as much strong beef tea as possible; the head was always shaved; and, in most, a blister was applied to the scalp.

Out of the whole eighteen cases, but one terminated fatally. The subject of it was very violently delirious on the day of her admission, and no account of her previous symptoms could be obtained. Death occurred on the third day afterwards. On making the autopsy, the brain was found to be slightly congested, and the grey matter of a darker color than usual. Peyer's patches in the small intestines were enlarged and very distinct, but not ulcerated. The spleen was enlarged, full of blood, and very soft; but all the other organs appeared to be in a normal condition.

Excluding, then, this fatal case, we will now examine the condition of the circulation in the remaining seventeen, more especially with regard to the influence of the treatment upon it. On the day that the administration of brandy, etc., was commenced, the pulse had, in five cases, a frequency of 136 per minute; in three, of 126; in seven, of from 120 to 126; and in one, of 116. After the measures above specified had been pursued for four days, the pulse had, in eight cases, fallen to 92; in five others it had fallen below 92 on the ninth day; and, in the remaining four, to below 90 on the sixth. Again, taking the day on which treatment was commenced as our starting point, the skin, previously hot and dry, relaxed, and became moist and perspirable, on the fifth day in nine cases; on the sixth day, in five cases; on the twelfth day, in one case; and in the remaining two the date of the crisis was not recorded.

The degree of success exhibited by the above facts is, we suspect, very considerably beyond that usually obtained in cases of so severe a type as those under consideration, and is very encouraging to a pursuance of a similar plan of treatment in future. That the success did really depend on the treatment, appeared to be conclusively evidenced in several cases, in which the pulse, progressively increasing in frequency up to the time that the brandy was ordered, steadily fell from that day forwards. The relapses of one or two, in consequence of the accidentally inefficient administration of the remedy, also afford important support to the same conclusion. In respect to the numerical age of the fever at which the brandy treatment was commenced, it varied so much in the different cases, that there does not appear to be any practical advantage in attempting to state it. In all, however, the first stage had passed, and low "typhus" symptoms had become fully developed. Dr. Todd is continuing the same plan of treatment on the fever patients now under his care, and hitherto with very pleasing results.—*Med. Times and Gazette.*

